# Diffuse erythema with “angel wings” sign in Belgian patients with antismall ubiquitin-like modifier activating enzyme antibody-associated dermatomyositis

**DOI:** 10.1016/j.jdcr.2025.01.043

**Published:** 2025-03-26

**Authors:** Anne-Catherine Dens, Jean-Baptiste Vulsteke, Margot Van Mechelen, Pieter Bourgeois, Julie Callens, Ellen De Langhe, Petra De Haes

**Affiliations:** a Department of Skeletal Biology and Engineering, KU Leuven, Leuven, Belgium; b Department of Dermatology, University Hospitals Leuven, Leuven, Belgium; c Department of Rheumatology, AZ Delta, Roeselare, Belgium; d Department of Rheumatology, University Hospitals Antwerp, Antwerp, Belgium; e Department of Dermatology, University Hospitals Antwerp, Antwerp, Belgium; f Department of Rheumatology, University Hospitals Leuven, Leuven, Belgium; g Department of Microbiology, Immunology and Transplantation, KU Leuven, Leuven, Belgium

**Keywords:** autoantibody, connective tissue disease, dermatomyositis

## Introduction

Inoue et al^1^ and Jia et al^2^ described the “angel wings” sign in Asian individuals with antismall ubiquitin-like modifier activating enzyme 1/2 (SAE1/2) antibody-positive dermatomyositis (DM). In this report, we present the observation of the “angel wings” sign in 2 European patients with DM carrying anti-SAE1/2 antibodies.

## Case report

**Fig 1:**
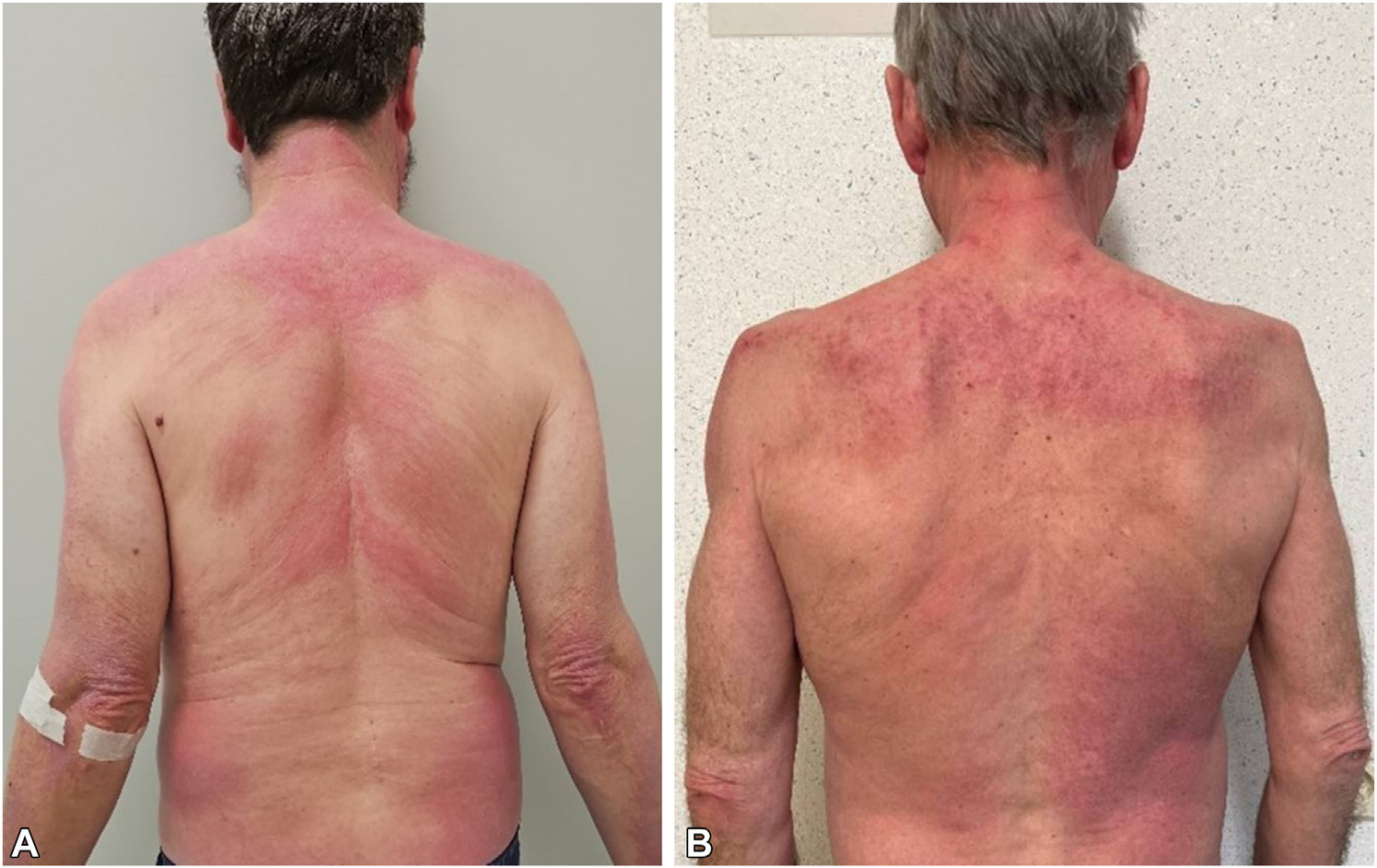
Diffuse erythema over the back with sparing of the scapular regions, resembling “angel wings,” in patients 1 (**A**) and 2 (**B**).

A 54-year-old man (case 1) presented with diffuse pruritic erythema that persisted for months. Dermatological examination revealed Gottron's papules, shawl- and V-sign, heliotrope edema and erythema, Gottron's sign on the elbows and knees, and diffuse erythema on the back with central sparing, consistent with the “angel wings” sign (Fig 1, *A*). Skin histopathology demonstrated a lichenoid junctional inflammatory infiltrate. The patient exhibited proximal muscle weakness without dysphagia, normal serum creatine kinase levels, but aberrant electromyography findings (smaller and polyphasic motor unit potentials in the iliopsoas and deltoideus muscles). Pulmonary function tests and thoracic computed tomography findings were normal. Antinuclear antibody testing was positive with a nuclear fine speckled pattern at a titer of 1/80, and anti-SAE1/2 antibodies were detected (D-tek immunodot assay, Belgium). Initial treatment with topical betamethasone dipropionate and oral prednisolone (1 mg/kg/day) was insufficient, necessitating adjunctive therapy with intravenous immunoglobulins (initiated at 2 g/kg over 3 days, followed by 1 g/kg/month) and methotrexate (17.5 mg/week). At present, cutaneous disease activity is decreasing, but the “angel wings” sign persists.

A 64-year-old male (case 2) presented with diffuse pruritic erythema, shawl and holster signs, Gottron's papules, and an “angel wings” sign on the back (Fig 1, *B*). Elevated creatine kinase level (maximum value 500 U/L, normal <190 U/L) and abnormal electromyography findings (early recruitment in the deltoid muscle with brief, small amplitude potentials) were compatible with active myositis. Pulmonary function test and computed tomography thorax were normal. Antinuclear antibody testing was positive with a homogenous pattern at a titer of 1/640 and anti-SAE1/2 antibodies. The patient was initially treated with topical betamethasone dipropionate and oral prednisolone (1 mg/kg/day) and methotrexate (25 mg/week). Rituximab was initiated because of an Epstein-Barr virus-positive lymphoproliferative disorder (4 cycles of 375 mg/m^2^/week, followed by 4 cycles once every 3 weeks). Due to persistent cutaneous and muscular disease activity, current treatment includes mycophenolate mofetil (2000 mg/day), hydroxychloroquine (400 mg/day), methylprednisolone (4 mg/day), and intravenous immunoglobulins (2 g/kg/month). Cutaneous disease activity is improving, with the “angel wings” sign fading.

At University Hospitals Leuven, 8 patients with DM have tested positive for anti-SAE1/2 antibodies. We performed a retrospective review of clinical records and available photographs for the 6 other patients with anti-SAE1/2 positivity. The “angel wings” sign was not observed in any of these patients, suggesting that this clinical marker is not consistently present in individuals with anti-SAE1/2 positive DM.

## Conclusion

The “angel wings” sign is a potential clinical indicator in patients with anti-SAE-positive DM beyond the Asian population. Further prospective documentation is necessary to establish its specificity and prevalence in different populations.

## Conflicts of interest

None disclosed.
